# Electrical Pulse Stimulation Protects C2C12 Myotubes against Hydrogen Peroxide-Induced Cytotoxicity via Nrf2/Antioxidant Pathway

**DOI:** 10.3390/antiox13060716

**Published:** 2024-06-12

**Authors:** Sarah Pribil Pardun, Anjali Bhat, Cody P. Anderson, Michael F. Allen, Will Bruening, Joel Jacob, Ved Vasishtha Pendyala, Li Yu, Taylor Bruett, Matthew C. Zimmerman, Song-Young Park, Irving H. Zucker, Lie Gao

**Affiliations:** 1 Department of Anesthesiology, University of Nebraska Medical Center, Omaha, NE 68198, USA; spribil@unmc.edu (S.P.P.); anjalibhatj@gmail.com (A.B.); wbruening@unmc.edu (W.B.); jjacob@unmc.edu (J.J.); vpendyala@wisc.edu (V.V.P.); 2 School of Health and Kinesiology, University of Nebraska Omaha, Omaha, NE 68182, USA; codypanderson@unomaha.edu (C.P.A.); michaelallen@unomaha.edu (M.F.A.); song-youngpark@unomaha.edu (S.-Y.P.); 3 Department of Cellular and Integrative Physiology, University of Nebraska Medical Center, Omaha, NE 68198, USA; lyu@unmc.edu (L.Y.); tbruett@unmc.edu (T.B.); mczimmerman@unmc.edu (M.C.Z.); izucker@unmc.edu (I.H.Z.)

**Keywords:** electrical pulse stimulation, mitochondria, ROS, Nrf2, antioxidant preconditioning, cellular protection

## Abstract

Skeletal muscle contraction evokes numerous biochemical alterations that underpin exercise benefits. This present study aimed to elucidate the mechanism for electrical pulse stimulation (EPS)-induced antioxidant adaptation in C2C12 myotubes. We found that EPS significantly upregulated Nrf2 and a broad array of downstream antioxidant enzymes involved in multiple antioxidant systems. These effects were completely abolished by pretreatment with a ROS scavenger, N-acetylcysteine. MitoSOX-Red, CM-H2DCFDA, and EPR spectroscopy revealed a significantly higher ROS level in mitochondria and cytosol in EPS cells compared to non-stimulated cells. Seahorse and Oroboros revealed that EPS significantly increased the maximal mitochondrial oxygen consumption rate, along with an upregulated protein expression of mitochondrial complexes I/V, mitofusin-1, and mitochondrial fission factor. A post-stimulation time-course experiment demonstrated that upregulated NQO1 and GSTA2 last at least 24 h following the cessation of EPS, whereas elevated ROS declines immediately. These findings suggest an antioxidant preconditioning effect in the EPS cells. A cell viability study suggested that the EPS cells displayed 11- and 36-fold higher survival rates compared to the control cells in response to 2 and 4 mM H_2_O_2_ treatment, respectively. In summary, we found that EPS upregulated a large group of antioxidant enzymes in C2C12 myotubes via a contraction-mitochondrial-ROS-Nrf2 pathway. This antioxidant adaptation protects cells against oxidative stress-associated cytotoxicity.

## 1. Introduction

Skeletal muscle (SkM) is a highly plastic tissue capable of functional and structural remodeling in response to numerous physiological stimuli. Particularly during endurance training, skeletal myocytes undergo various challenges from neuronal, hormonal, mechanical, and metabolic stressors, leading to genetic and biochemical adaptations that ultimately determine the beneficial phenotypes evoked by chronic exercise [1].

A predominant stressor on contracting muscle fibers is reactive oxygen species (ROS). ROS, at high levels, can react with biomolecules to oxidize proteins, lipids, and nucleic acids. This results in cell damage and organ dysfunction, a pathological redox state referred to as “oxidative stress”, which underlies the pathogenesis of numerous diseases. Indeed, in skeletal muscle, it is well-known that excessive ROS contributes to muscle fatigue during exhaustive exercise [2], doxorubicin (DOX)-induced muscle wasting [3], and an age-related progressive loss of muscle mass and strength [4]. On the other hand, ROS at low- to moderate- levels function as essential intracellular signaling molecules, playing a crucial role in many biological processes under normal conditions. Physiological ROS can induce post-translational modifications (PTMs) on specific target proteins by reversible oxidation of sulfur groups and metal centers [5], therefore altering protein activity, localization, and interactions that result in the orchestration of various biological events in cells and organs, such as cell proliferation, differentiation, migration, and angiogenesis [6]. Physiological ROS, or “normal redox signaling,” is essential for maintaining normal metabolism and function of cells and organs [7]. Indeed, studies have demonstrated that selective depletion of ROS from normal skeletal muscle decreases force generation [8]. Accordingly, there is an optimal ROS concentration in skeletal myocytes, which determines the maximal force generation during contraction. Deviations of redox status away from this point, either via an increase or decrease in ROS concentration, will impair muscle contractility, forming a classic bell-shaped hormesis dose–response curve [9].

While there is no clear boundary of ROS levels to induce oxidative stress and function as physiological redox signaling, there is no doubt that these free radicals can activate intracellular antioxidant defense under both conditions via compensatory mechanisms to upregulate enzymatic and non-enzymatic antioxidants. These enhanced antioxidant mechanisms not only support the cells to overcome instant oxidative stress but also provide cells with an extended antioxidant capability to counteract subsequent similar insults, a phenomenon we term “antioxidant preconditioning”. This process has been proposed as a foundation of redox adaptation of skeletal muscle to endurance exercise [9].

A well-known sensor-effector apparatus in response to a redox disturbance is the Keap1-Nrf2 system. Keap1 acts as a ROS sensor and Nrf2 repressor, while Nrf2 is responsible for activating multiple antioxidant genes, thus playing a critical role in maintaining intracellular redox homeostasis [10]. By employing muscle-specific Nrf2 deletion and overexpression transgenic mouse models, iMS-*Nrf2^flox/flox^* and iMS-*Keap1^flox/flox^*, we previously identified over 200 cytoprotective and regulative proteins governed by Nrf2 in skeletal muscle, which are involved in several intracellular signaling pathways. These include antioxidant defense and mitochondrial function [11]. We further demonstrated that these Nrf2-orchestrated molecular networks contribute to chronic exercise-induced enhancement of contractility and muscle antioxidant adaptation [12]. However, it remains to be determined if short-term endurance training (STET) can evoke similar beneficial effects on skeletal myocytes to protect the cells against oxidative stress-associated injury.

In the present study, we used electrical pulse stimulation (EPS) on differentiated C2C12 myotubes, a validated in vitro and widely used acute muscle preparation [13,14], to address the hypothesis that a short-term contraction can evoke an antioxidant preconditioning in skeletal myocytes via a mitochondrial-ROS-Nrf2 pathway. Furthermore, we provide evidence to demonstrate that this antioxidant adaptation protects cells against H_2_O_2_-induced toxicity.

## 2. Materials and Methods

### 2.1. Cell Culture

The C2C12 cell line (CRL-1722, ATCC, Manassas, VA, USA) was used in these experiments. Cells were seeded in a 6-well plate at ~50,000 or 100,000 cells per well and grown for one day in complete media (CpM) (Dulbecco’s Modification of Eagle’s Medium (DMEM/HIGH GLUCOSE; HyClone, Logan, UT, USA) supplemented with 10% fetal bovine serum (FBS; Gibco/ThermoFisher Scientific New Zealand Limited, Auckland, New Zealand) and 1% penicillin/streptomycin (P/S)). After ~24 h, the media was changed from CpM to differentiation media (DM) (DMEM supplemented with 5% horse serum (HS; Gibco/ThermoFisher Scientific) and 1% P/S), where the cells differentiated from myoblasts to myotubes after four days in culture at 37 °C, 5% CO_2_, 20% O_2_, and 95% humidity. On the fourth day, the media were replaced by fresh DM, and the cells underwent electrical pulse stimulation (EPS).

### 2.2. Electrical Pulse Stimulation (EPS) and Cell Collection

The electrical current of EPS was delivered to the C2C12 myotubes via stimulation electrodes designed and fabricated in our lab. These electrodes were built on the lid of standard 6-well plates, similar to what has previously been reported [15]. On the first day of EPS, the regular 6-well plate lids were replaced by the EPS lids containing the electrodes, which were connected to a single-channel pulse generator (A310 Accupulse, WPI, Sarasota, FL, USA). The EPS lids were cleaned before and after each study using the following protocol: The EPS chamber was flooded with 70% ethanol for 10 min. The electrodes and EPS lid were washed with ~3 mL of sterile PBS. Finally, the EPS lid was exposed to UV light for 10–30 min in the cell-culture laminar flow hood. The C2C12 myotubes underwent EPS at 10 v, 50 Hz, 10 ms, 0.3 s/3 s for 1 h/day for 4 days. At the end of the EPS, the cells were rinsed with 200 µL of sterile PBS, followed by trypsinizing with 200 µL of 0.05% trypsin for 2 min at 37 °C. The cells were then collected and transferred to 1.5 mL EP tubes, which were stored at −80 °C for future analysis.

### 2.3. Western Blotting

#### 2.3.1. Sample Preparation (Protein Extraction)

Cells were defrosted from −80 °C, incubated on ice in 100 µL of 100:1 RIPA:PIC for 15 min, and then homogenized utilizing a sonicator. The soluble proteins of cell lysate were then extracted by centrifugation for 20 min at 13,800× *g* and 4 °C. The protein concentration was measured via BSA assay and a Multiskan Sky Microplate Spectrometer (Thermo Scientific Multiskan FC, Singapore). Samples were then diluted to 2 µg/µL with RIPA. An equal volume of 2X SDS was added to each sample to further dilute the samples to 1 µg/µL and 1X SDS. The samples were then boiled in a 70 °C water bath for 2 min and immediately returned to ice.

#### 2.3.2. Gel Electrophoresis

The protein samples (15 μg in 15 μL/each sample) were loaded on a precast polyacrylamide gel (NW00105BOX, Invitrogen, Carlsbad, CA, USA) along with 2 μL of Invitrogen MagicMark™ Western Standard and 4 μL of Bio Rad Precision Plus Protein Dual Color Standards in separate wells. Electrophoresis was performed using a Mini Gel Tank and Blot Module Set (NW2000, Invitrogen), initially at 70 V, for approximately 15 min, and then at 120 V until the desired separation had been reached.

#### 2.3.3. Membrane Transfer (iBlot2)

The fractionated protein on the gel was electrically transferred onto a nitrocellulose membrane by employing Thermo Fischer Scientific iBlot2 (IB21001, Thermo Fisher Israel Ltd, Kiryat Shmona, Israel) with the preset program P0 (20 V for 1 min, then 23 V for 4 min, followed by 25 V for 2 min). The membranes were gently rinsed with Milli-Q water and then stained with Ponceau S for 5 min to visualize the total protein. Once ideal bands of the total protein were obtained, Ponceau S on the membrane was removed by rinsing it in 10 mL of 1X phosphate-buffered saline-Tween 20 solution (PBST) for 5 min.

#### 2.3.4. Immunodetection (Antibodies)

The membranes were then blocked for 30 min in 5% milk in 1X PBST at room temperature; this was followed by two 5 min washes in 10 mL of 1X PBST at room temperature. The membranes were then incubated in primary antibody in 4% BSA overnight at 4 °C. The primary antibodies used in the present experiment were purchased from Abcam (ab-), ABclonal (A-), Santa Cruz Biotechnology (sc-), and Proteintech (-AB/-ig), including NQO1 (ab80588), GSTA2 (ab232833), GSTA4 (ab231601), GPX1 (ab22604), PRX Pathway (TRX, TXNRD1, and PRX1; ab184868), total OXPHOS (ab110413), calmodulin 1/2/3 (A4885), MFN1 (A9880), MFF (A8700), DRP1 (A2586), FIS1 (A21527), SOD1 (sc-8637), SOD2 (sc-30080), catalase (sc-50508), MyoG (sc-576), Nrf2 (A0674 and 16396-1-AP), and mTOR (66888-1-ig). The following day, the membranes were washed three times for 5 min in 10 mL of 1X PBST; this was followed by incubation in anti-rabbit IgG horseradish peroxidase secondary antibody (1:5000) in 5% milk in 1X PBST for 30 min at room temperature. After this, the membrane was again washed three times for 5 min in 10 mL of 1X PBST. The membranes were developed in the chemiluminescence detection reagent (SuperSignal West Dura or SuperSignal Femto; Thermo Scientific, Rockford, IL, USA). The films were scanned using the G:Box Syngene and Genesys. The intensity of the bands was quantified using the ImageJ software (version number 1.52a, NIH) and normalized for loading using Ponceau S staining.

### 2.4. ROS Measurements and NAC Application

#### 2.4.1. Electron Paramagnetic Resonance (EPR) Spectroscopy

C2C12 myoblasts were seeded in six-well plates (100,000 cells/well), followed by differentiation and the EPS processes, as described above. After the final EPS, the old DM was replaced with fresh DM, and the cells were processed for EPR spectroscopy analysis, as described below at their specified time points (0, 3, 12, or 24 h after EPS or NS).

The media were removed from the wells, and the cells were then washed twice with 1 mL of KDD buffer (Krebs-HEPES buffer [pH 7.4]; 99 mM NaCl, 4.69 mM KCl, 2.5 mM CaCl_2_, 1.2 mM MgSO_4_, 25 mM NaHCO_3_, 1.03 mM KH_2_PO_4_, 5.6 mM d-glucose, 20 mM HEPES, 5 μM diethyldithiocarbamic acid sodium salt [DETC], and 25 μM deferoxamine). Then, the cells were mixed with 200 μM cell-permeable ROS-sensitive spin probe 1-hydroxy-3-methoxycarbonyl-2,2,5,5-tetramethyl pyrrolidine (CMH; Noxygen Science Transfer and Diagnostics, Elzach, Germany) and incubated for 1 h and 15 min at 37 °C. After incubation, 900 µL of the KDD buffer was removed from the wells, and the cells were gently scraped into the remaining 100 µL. A total of 50 µL of the cell suspension was pipetted into an EPR glass capillary tube and inserted into a Bruker e-scan EPR spectrometer with the following settings: field sweep width, 60.0 G; microwave frequency, 9.75 kHz; microwave power, 21.90 mW; modulation amplitude, 2.37 G; conversion time, 10.24 ms; and time constant, 40.96 ms. The remaining cell suspension was utilized to count the number of cells to normalize the EPR spectrum amplitude.

#### 2.4.2. Confocal Imaging—MitoSOX Red (MSR, Mitochondrial ROS Indicator) and CM-H2DCFDA (Cytoplasmic ROS Indicator)

C2C12 myoblasts were seeded in six-well plates (5000 cells/well) containing polylysine-coated slides for cell growth, followed by differentiation and the EPS processes, as described above. Immediately after the final day of EPS, the well was washed with 1 mL of HBSS, and the coverslips were then transferred to a 24-well plate, where 2 mL of warm 1 µM MSR working solution or 1 mL of 5 µM CM-H2DCFDA was added, followed by incubation at 37 °C and 5% CO_2_ for 30 min. The coverslips were then transferred into clean black boxes and gently washed 3 times with warm HBSS. The cells on the coverslips were fixed with cold 4% paraformaldehyde (PFA) for 10 min, washed 3 times with PBS, and then mounted onto a glass slide using mounting medium (sc-24941 with DAPI) and clear nail polish. The fluorescence was analyzed via confocal microscopy at the following wavelengths: Mitochondrial ROS: Ex 488 nm/Em 550–650 nm; Total ROS: Ex 488 nm/Em 520 nm; and Cellular Nuclear: Ex 360 nm/Ex 460 nm.

#### 2.4.3. N-Acetylcysteine (NAC) Treatment

Three six-well plates were seeded at 100,000 cells per well and underwent differentiation as described above. On the fourth day of differentiation, 40.80 mg of NAC was dissolved in 1 mL of the CpM, which was then further diluted to a final working concentration of 5 mM NAC in 50 mL of the DM. The pH of this medium was neutralized to ~7.5 with 0.5 M NaOH and then filtered through a 0.2 µm syringe filter. After 15 h of pretreatment with this DM containing 5 mM NAC, the cells underwent EPS for 1 h at 50 Hz, 10 V, 10 ms, and 0.3 s/3 s. Eight hours after EPS, the cells underwent a second treatment of NAC following the protocol outlined above. This was repeated daily for four days. After the fourth and final day of EPS, the cells were collected as described above.

### 2.5. Mitochondrial Function Assays

#### 2.5.1. Seahorse—Mito Stress Test

After undergoing 4 days of EPS, the cells were collected as described above and replated in 96-well plates at 10,000 cells per well in 100 μL of DM. The sensor cartridge was hydrated in a calibration buffer at 37 °C in a non-CO_2_ incubator overnight. Sterile-filtered 10 mM glucose, 2 mM glutamate, and 1 mM pyruvate were added to the Seahorse medium, whose pH was then corrected to ~7.4 with 1 M HCl or 0.5 M NaOH. Next, 80 μL of DM was removed from each well of the XF96-well plate, followed by washing with 100 μL of Seahorse medium. A total of 160 μL of fresh Seahorse medium was added to each well, bringing the final volume to 180 μL. The cells were incubated in the Seahorse medium in a non-CO_2_ incubator for 1 h. Working solutions of Oligomycin, FCCP, and Rotenone/Antimycin A were made and then added to the injection ports of the sensor cartridge in the respective order. The plate was run on a 96-well plate Seahorse instrument using Wave programming. The Seahorse XF Mito Stress Test Report Generator was used to retrieve the data for later analysis.

#### 2.5.2. Oroboros–High-Resolution Respirometry

After undergoing 4 days of EPS or no stimulation (controls), approximately 300,000 cells suspended in DM were centrifuged at 1500× *g* for 5 min, and the cell pellet was resuspended in 2 mL of mitochondrial respiration medium [MiR05, containing 0.5 mM EGTA, 3 mM MgCl_2_, 60 mM lactobionic acid, 20 mM taurine, 10 mM KH_2_PO_4_, 20 mM HEPES, 110 mM D-Sucrose, 1 g/L BSA, pH 7.1] in a high-resolution respirometer (Oxygraph-2k, Oroboros, Innsbruck, Austria) to measure the mitochondrial respiratory function. The chamber block maintained a constant temperature of 37 °C and magnetic stir bars mixed the solution continuously at 750 RPM. The chambers were hyper-oxygenated to 300 μM O_2_ via a syringe with pure oxygen from an Oxia device (Oxia, Innsbruck, Austria). After the O_2_ respiration rate stabilized, the cells were permeabilized with digitonin (4.05 μM) for 20 min. After permeabilization, substrates were administered in the following order: (1) malate (2 mM) and glutamate (10 mM), (2) adenosine diphosphate (ADP, 5 mM), (3) succinate (10 mM), (4) cytochrome c (10 μM), (5) rotenone (0.5 μM), (6) oligomycin (5 nM), (7) antimycin-A (2.5 μM), and (8) ascorbate (2 mM) and N,N,N’,N’-tetramethyl-p-phenylenediamine dihydrochloride (TMPD, 0.5 mM) [16]. Mitochondrial complexes were assessed as follows: (1) complex I state 2 (LEAK respiration) was assessed after malate and glutamate, (2) complex I state 3 was assessed after malate and glutamate + ADP, (3) complex I + II state 3 was assessed after malate and glutamate + ADP + succinate, (4) the membrane integrity was evaluated after the addition of cytochrome c, (5) complex II state 3 was assessed after the addition of rotenone, and (6) complex IV respiration was assessed after ascorbate and TMPD. The respiration rate for each substrate or inhibitor was recorded after the O_2_ flux reached a stable value (~5 min for substrates and ~10 min for inhibitors). The O_2_ respiration rates were measured as picomoles of O_2_ per second per mL and were converted to attomoles of O_2_ per second per cell (amol·s-1·cell).

### 2.6. H_2_O_2_ Treatment and CCK-8 Assay

After undergoing 4-day EPS, the cells were collected as described above and replated in 96-well plates at 5000 cells per well in 100 μL of CpM. The cells were allowed to adhere to the plate overnight. After the cells had adhered to the plate, the CpM was removed and replaced with 100 μL of PBS in CpM (control) or 2 or 4 mM H_2_O_2_ in CpM. The cells were incubated in H_2_O_2_ at 5% CO_2_ and 37 °C for 5 h. After 5 h, 10 μL of CCK-8 was added to each well. The cells were incubated in CCK-8 at 5% CO_2_ and 37 °C for 1 h. The absorbance was measured by a Multiskan Sky Microplate Spectrometer at 450 nm to evaluate cell viability.

### 2.7. Statistical Analyses

Data are expressed as means ± SD. A t-test was used for analyzing the differences in protein expression between the EPS and the control group using GraphPad Prism 8 software. A two-way ANOVA was used to analyze the EPS-NAC data shown in panels C and D of Figure 4. Tukey’s honestly significant difference test was performed after ANOVA analysis. A *p*-value of <0.05 was taken as indicative of the statistical significance.

## 3. Results

### 3.1. EPS Evokes Voltage- and Time-Dependent Upregulation of NQO1 and GSTA2 Proteins

**Figure 1:**
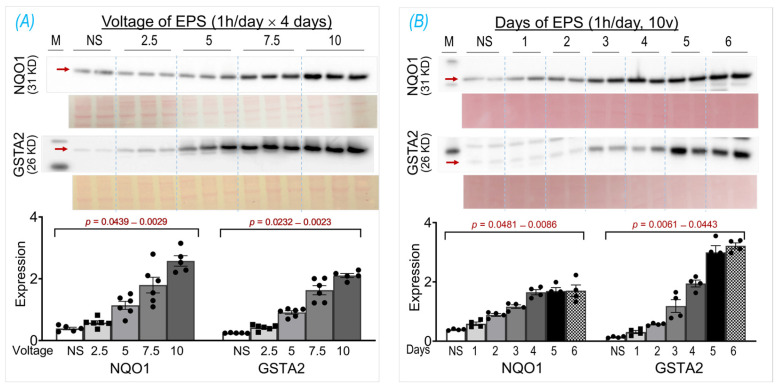
NQO1 and GSTA2 protein expression in C2C12 myotubes receiving EPS at different voltages (**A**) and durations (**B**). Red arrows indicate the target bands. Data are expressed as mean ± SD; *n* = 4–6/each group. NS, non-stimulated control. The y-axes of the bar graphs indicate protein expression normalized by Ponceau S.

The basic EPS parameters (10 ms, 50 Hz, 0.3 s/3 s) were adopted from our previous experiments in mice, where EPS was employed to induce an in situ tetanic contraction for evaluating the SkM fatigue development and force generation of mice with chronic heart failure [17], Nrf2 gene manipulation [11], and chronic exercise training [12]. The frequency of EPS was set as 50 Hz because mouse motor-unit output impulses fall within the range of 30–80 Hz [18]. To optimize the EPS suitable for the present study in cultured cells, we investigated the protein expression of NQO1 and GSTA2, two primary targets of Nrf2 in SkM [11], in response to the EPS administration at different voltages (2.5–10 V; Panel A of Figure 1) and different durations (1–6 days; Panel B of Figure 1). Clearly, both NQO1 and GSTA2 were upregulated by EPS in a voltage- and duration-dependent manner, with a minor response difference between these two proteins. As shown in Panel A, 10 V-EPS evoked a maximal expression in both, whereas 2.5 V-EPS upregulated the expression only in GSTA2 but not in NQO1. As shown in Panel B, NQO1 displayed a maximal response to EPS on day 4, while GSTA2’s maximal expression existed on day 5. Based on these results, we chose the EPS parameters of 10 ms, 50 Hz, 0.3 s/3 s, 10 V, and 1 h/day for 4 days for all of the following experiments.

### 3.2. EPS Increases Nrf2 Protein Content in C2C12 Myotubes

**Figure 2:**
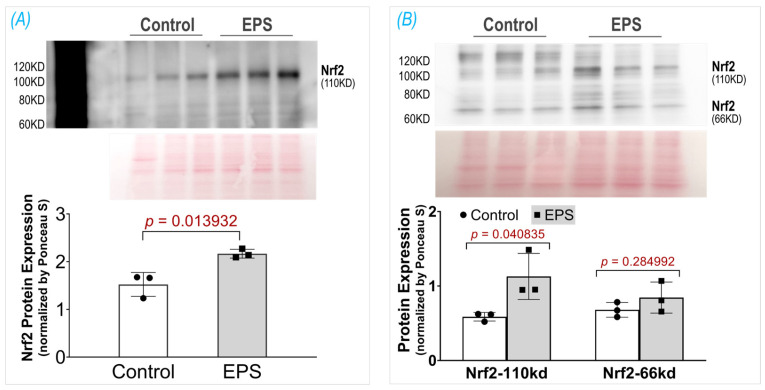
Protein expression of Nrf2 in the C2C12 myotubes receiving EPS, detected by using two different antibodies: (**A**) A0674 (ABclonal) and (**B**) 16396-1-AP (Proteintech). Data are expressed as mean ± SD; *n* = 3/each group.

In two previous experiments from our laboratory, we employed the ab13550 antibody (AbCam) to probe Nrf2 protein in mouse SkM lysate and showed a single specific band at 110 kDa, which was significantly upregulated by administrating the Nrf2 activator curcumin [19] or by deleting the Keap1 gene [11]. Unfortunately, the current lot of ab13550 did not detect a Nrf2 band in mouse muscle tissue nor in C2C12 myotubes. We therefore tested seven other Nrf2 antibodies provided by different vendors, including AbCam (ab62352), Proteintech (16396-1-AP, 66504-1-lg), ABclonal (A0674, A1244, A11159), and Invitrogen (PA5-67520). Among these antibodies, we found that two antibodies, A0674 and 16396-1-AP, detected a specific Nrf2 band at 110 kDa, whose intensity was significantly higher in the EPS cells than in the control cells, as shown in Figure 2. Interestingly, in the blot of 16396-1-AP antibody, we found an additional Nrf2 band at 66 kDa, which was not changed by the EPS, as shown in Panel B. The different blots of these two antibodies may reflect a different immunogen used to make the antibodies where the NP_006155.2 was used for A0674 (ABclonal) and the AG9489 (Proteintech) for the 16396-1-A. Since the predicted molecular weight of Nrf2, based on its amino acid sequence, is ~66 kDa, we think that the band at ~66 kDa represents a non-modified and non-activated Nrf2 protein, whereas the band in ~110 kDa is the biologically relevant and activated Nrf2, whose molecular weight is higher due to post-translational modifications (PTMs) such as acetylation, phosphorylation, ubiquitination, and SUMOylation [20]. The observation and interpretation of the Nrf2 blots showing two bands have been discussed by two laboratories in the field [21,22]. Although the current data clearly showed an increased Nrf2 protein content in whole-cell lysates of EPS-C2C12 myotubes, the underlying molecular mechanisms remain to be elucidated. We postulated that both the activation of the Nrf2 gene (NFE2L2) and reduction of the proteasome-mediated Nrf2 protein degradation contribute to this increased Nrf2 protein content, with the latter mechanism being the predominant. This conclusion is based on the data shown in Figure 4, where we found that EPS dramatically elevated intracellular ROS, the principal mediator to dissociate Nrf2 from Keap1, and that pretreatment with the ROS scavenger, N-acetylcysteine (NAC), completely abolished the EPS-induced increase in Nrf2 protein.

### 3.3. EPS Upregulates Multiple Antioxidant Proteins

**Figure 3:**
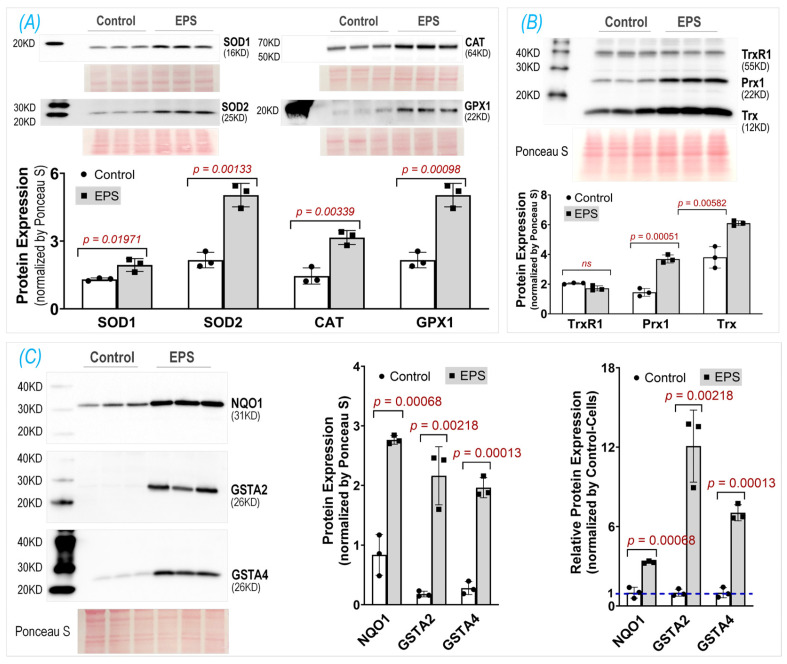
Protein expression of key antioxidant components in three antioxidant systems: the first-line antioxidant system (**A**), thioredoxin antioxidant system (**B**), and glutathione antioxidant system (**C**). The middle subpanel of (**C**) shows raw data; the right subpanel of (**C**) is relative data normalized by the expression in the control group. Data are expressed as mean ± SD; *n* = 3/each group.

After demonstrating increased Nrf2 protein in the EPS cells, we then evaluated the Nrf2 downstream target proteins that constitute cellular antioxidant defenses. We found that, in the EPS cells, a large group of proteins associated with multiple antioxidant systems were significantly upregulated (Figure 3). These proteins include the components of the first-line antioxidant system (SOD1, SOD2, Cat, and GPX1; Panel A), the thioredoxin antioxidant system (Trx and Prx1; Panel B), and the glutathione antioxidant system (GSTA2, GSTA4, and NQO1; Panel C). Among these evaluated Nrf2 target proteins, NQO1, GSTA2, and GSTA4 were the top three most upregulated proteins following EPS, which were 3-, 12-, and 7-fold higher in the EPS cells than the control cells, respectively (right subpanel of Panel C). These data suggest that EPS can activate multiple antioxidant systems in C2C12 cells, where the glutathione system is the most mobilized.

### 3.4. EPS-Evoked Activation of Nrf2/Antioxidant System Relies on ROS

**Figure 4:**
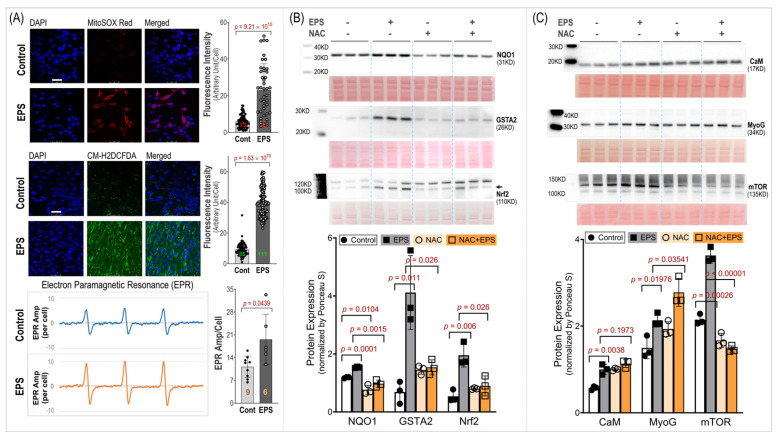
ROS level and its role in EPS-induced protein expression. (**A**): ROS levels in mitochondria (MitoSOX Red), cytosol (CM-H2DCFDA), and whole cells (EPR). Left subpanels: representative confocal images (scale bar: 50 μm) and EPR spectra. Right subpanels: quantitative fluorescence intensity and EPR results. The red, green, and orange numbers within the bars indicate cell and sample numbers. (**B**,**C**): Protein expression of Nrf2/antioxidant enzymes and non-antioxidant proteins. Data are expressed as mean ± SD; *n* = 3/each group. NAC: N-acetylcysteine; 5 mM.

To investigate the role of ROS in the EPS-evoked activation of Nrf2/antioxidant systems, we employed three techniques to measure the ROS levels and used a ROS scavenger, N-acetylcysteine (NAC). As can be seen in the MitoSOX-Red, CM-H2DCFDA, and EPR spectroscopy data shown in Panel A of Figure 4, the EPS cells exhibited a significantly higher fluorescence intensity and EPR spectrum amplitude compared to the control cells. These data indicate a significantly high ROS level in the mitochondria, cytosol, and whole cells of the C2C12 myotubes that received stimulation and demonstrated an increased ROS generation from the mitochondria of the contracting muscle. Panel B of Figure 4 shows that the EPS-induced upregulation of Nrf2/antioxidant enzymes was completely abolished by the NAC pretreatment (EPS + NAC), suggesting that the increased ROS is the exclusive mediator of the Nrf2/antioxidant upregulation induced by EPS. Interestingly, we found that the NQO1 expression was significantly lower in the cells treated with NAC alone compared to the cells without any treatment, suggesting that the basal expression of this antioxidant enzyme relies on the physiological levels of ROS. Panel C of Figure 4 shows the expression of three non-antioxidant proteins, where NAC had no effects on the EPS-induced upregulation of CaM and MyoG but did abolish the mTOR response.

### 3.5. EPS Enhances Mitochondrial Oxidative Phosphorylation and Dynamics

**Figure 5:**
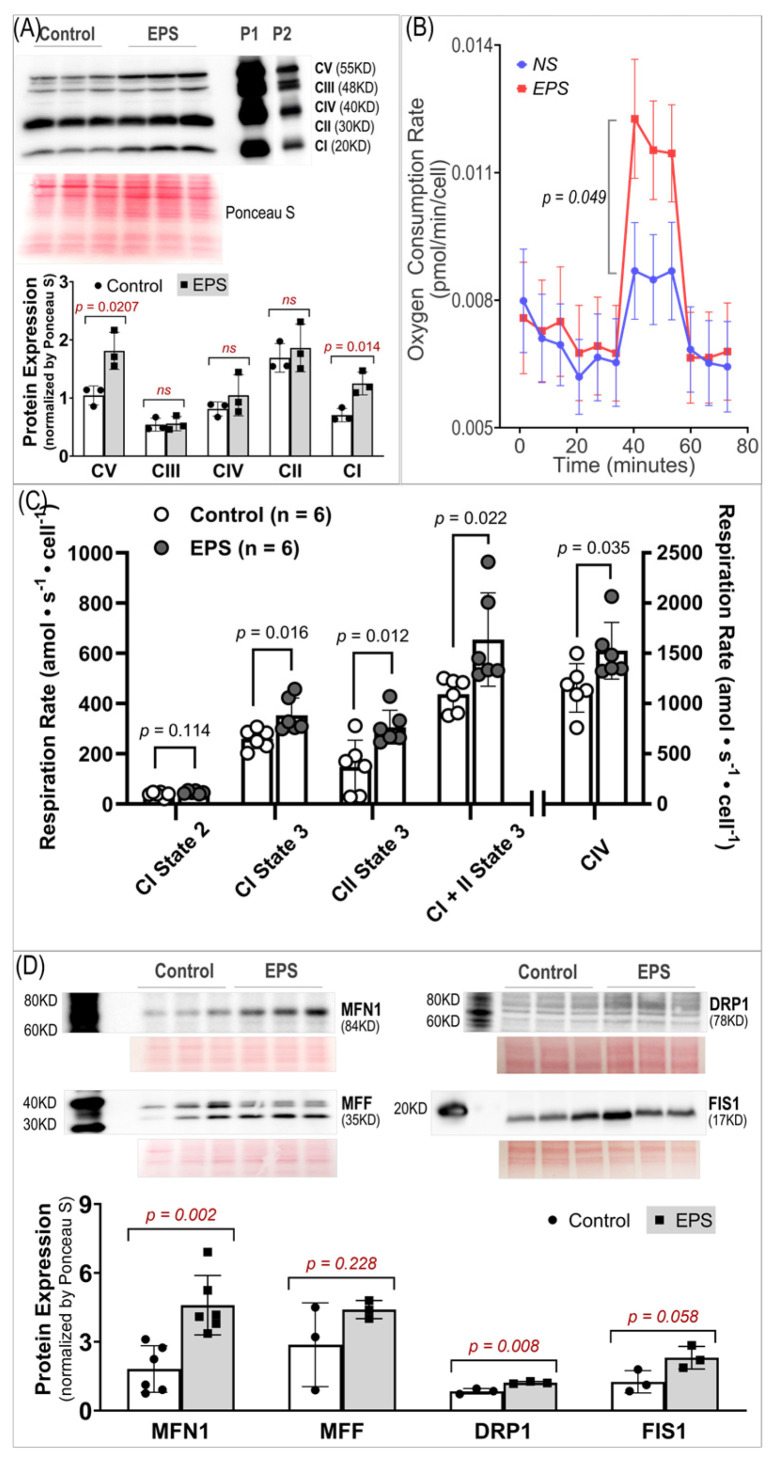
Mitochondrial protein expression and function. (**A**) Protein expression of respiratory complexes I–V. *n* = 3/group. (**B**) Oxygen consumption rate measured by Seahorse. *n* = 46 in the NS group and 46 in the EPS group. (**C**) Oxygen consumption rates measured by Oroboros. *n* = 6/group. (**D**) Protein expression of fission/fusion factors. *n* = 3/group. All data are expressed as mean ± SD. ns, not significant.

Given that mitochondria are one of the major sources of ROS in skeletal myocytes during contraction, we examined the effects of EPS on the oxidative phosphorylation (OXPHOS) complex protein expression, oxygen consumption rate, and protein expression of four factors associated with mitochondrial dynamics. We found that EPS significantly upregulated CI, the main site of ROS production [23], and CV, the ATP synthase [24], whereas the CII, CIII, and CIV expression were left unchanged (Panel A, Figure 5). Correspondingly, the FCCP-mediated uncoupled maximal oxygen consumption rate was significantly increased in the EPS cells compared to the control cells (Panel B, Figure 5). Furthermore, ADP-stimulated respiration (state 3) was increased in the EPS compared to non-stimulated cells through complex I, complex II, and complex I + II (Panel C, Figure 5). In addition, complex IV respiration was elevated, and complex I state 2 respiration was unchanged in the EPS compared to non-stimulated cells (Panel C, Figure 5). Overall, these data suggest that EPS can mediate robust increases in OXPHOS capacity in C2C12 myotubes by upregulating CI and CV expression. Mitochondria are highly dynamic cytoplasmic organelles. During contraction, the mitochondrial integrity and homeostasis in SkM have to be maintained through continual fusion and fission [25,26]. Therefore, we evaluated the expression of four proteins associated with mitochondrial dynamics and found that EPS upregulated MFN1 and DRP1 but had no effect on MFF and FIS1 (Panel D, Figure 5).

### 3.6. EPS Evokes Antioxidant Preconditioning to Protect Cells against H_2_O_2_-Induced Injury

**Figure 6:**
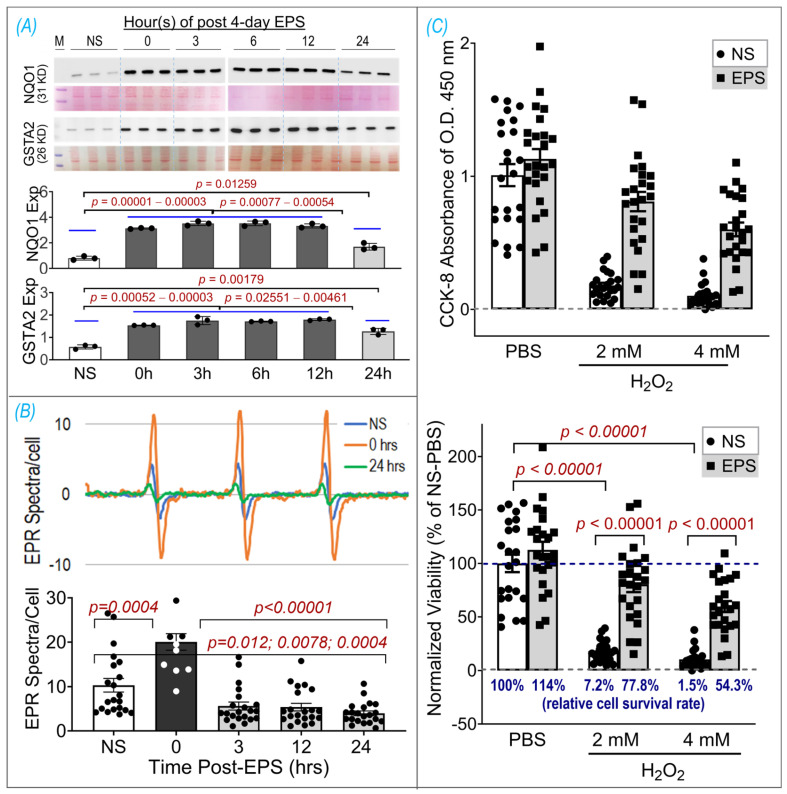
EPS-induced antioxidant preconditioning and protective effects. (**A**) Time course of NQO1 and GSTA2 expressions, which were normalized by Ponceau S. (**B**) Time course of ROS levels. (**C**) Cell viability assay using CCK-8 shown as raw data of OD 450 nm absorbance (upper panel) and normalized viability by NS-PBS as a percentage (lower panel). Data are expressed as mean ± SD; *n* = 3/group in (**A**), 12–21/group in (**B**), and 23–24/group in (**C**). NS, non-stimulation.

To determine how long antioxidant proteins can remain upregulated after stopping stimulation, we carried out an experiment to evaluate the time course of NQO1 and GSTA2 protein expression in the samples harvested at 0, 3, 6, 12, and 24 h after EPS (Panel A of Figure 6). We did not find a significant difference in protein expression between the samples at 0, 3, 6, and 12 h. However, protein expression in the 24 h sample was significantly lower than that in other EPS samples but still significantly higher than that in the non-stimulated control sample. These data suggest that EPS-enhanced antioxidant enzyme expression can last at least 24 h. On the other hand, the EPR spectroscopy showed a significant increase in ROS immediately after EPS (i.e., 0 h), which rapidly declined in the 3–24 h samples to levels below that in the non-EPS sample (Panel B, Figure 6). These data suggest that the upregulated endogenous antioxidants by EPS provide C2C12 myotubes with excess antioxidant capacity, a status we term “antioxidant preconditioning”, which can protect the cells against subsequent oxidative stressor-induced injury. As can be seen in Panel C of Figure 6, the survival rates of the EPS cells exposed to 2 mM and 4 mM H_2_O_2_ were 77.8% and 54.3% of the PBS-NS control group, about 11- and 36-fold higher than that of the H_2_O_2_-NS cells, whose survival rates were 7.2% and 1.5%, respectively.

## 4. Discussion

Using EPS on cultured C2C12 myotubes to investigate the effects of contraction on the intracellular antioxidant system in SkM, we showed that EPS for up to 4 days induced an obvious time- and dose-dependent upregulation of NQO1 and GSTA2 protein expression. We found that EPS markedly activated Nrf2 and multiple antioxidant systems, including the first-line antioxidant system, the thioredoxin system, and the glutathione system, by upregulating a large group of antioxidant enzymes and antioxidant-associated proteins. Among these systems, the glutathione system displayed the largest response to EPS, with approximately a 10-fold increase in GSTA2 and GSTA4 protein expression (Panel C, Figure 3). We further demonstrated that the EPS-mediated increase in ROS played a critical role in the activation of Nrf2/antioxidant systems since EPS-mediated enhancements in antioxidant capacity were abolished when the C2C12 myotubes were pre-treated with the ROS scavenger, NAC (Panel B, Figure 4).

Importantly, our data of substantially elevated mitochondrial-derived superoxide after EPS supports the view that mitochondria may be one of the major sources of ROS and is responsible for the activation of Nrf2/antioxidant pathways (Panel A, Figure 4). Furthermore, our data suggest that EPS-induced mitochondrial adaptations may promote EPS-induced augmentation in antioxidant capacity. Of note, complex IV respiration was significantly increased after 4 days of EPS, compared to the non-stimulated cells (Panels B and C, Figure 5). Furthermore, ADP-stimulated respiration (state 3) through complex I, complex II, and complex I + II were all elevated after 4 days of EPS (Panel C, Figure 5). In addition, these adaptations were paralleled by an increased protein expression of complexes I—the main mitochondrial site for ROS production [27]—and V (Panel A, Figure 5) and upregulated fission/fusion factors (Panel D, Figure 5). Altogether, these data indicate that EPS leads to substantial adaptations to mitochondrial function and elevations in OXPHOS capacity, which is consistent with the known adaptations in human SkM mitochondria after aerobic training [28,29]. Importantly, the stepwise elevation in OXPHOS capacity due to repeated EPS exposure may also lead to elevations in mitochondrial ROS production during high-intensity EPS [30,31,32], which may provide an incremental stimulus necessary to induce cumulative increases in antioxidant capacity through Nrf2, as observed in the present study (Panel A, Figure 1). Furthermore, our data support the notion that ROS is transiently elevated during EPS (Panel B, Figure 6) while the antioxidant capacity remains elevated up to 24 h after EPS (Panel A, Figure 6), indicating an overcompensation of antioxidant synthesis and the development of an antioxidant reserve. Importantly, we demonstrated that “antioxidant preconditioning” induced by EPS protects the C2C12 myotubules from future oxidative injury since the EPS cells were more resistant to H_2_O_2_-induced cytotoxicity than the non-stimulated control cells. Overall, our data indicate that mitochondrial ROS during EPS activates the Nrf2/antioxidant pathways and may lead to an antioxidant reserve that protects cells from future oxidative injury.

C2C12 is a frequently used cell line to investigate the physiology and pathology of skeletal myocytes. After exposure to a differential medium, C2C12 are transformed from primitive myoblasts into mature myotubes, which have the primary characteristics of rodent SkM, including the ability to contract and generate force. Indeed, it has been demonstrated that EPS evokes the actual contraction of C2C12 myotubes, making this cell line a convenient in vitro model that is suitable for investigating the physiological significance and molecular responses of skeletal myocytes to stimulated exercise [14]. A single bout of EPS at 1 Hz for 1, 3, or 6 h duration has been demonstrated to upregulate the Nrf2/antioxidant system in C2C12 myotubes [33]. We employed EPS at 50 Hz and 1 h per day for 4 consecutive days in the present experiment, which may better simulate the SkM response to chronic short-term exercise. Importantly, our time-course data clearly show a significantly higher protein expression of NQO1 and GSTA2 in 4-day-EPS-cells compared to those that undergo 1 day of EPS. These findings suggest cumulative effects of repeated contraction on the activation of the muscle antioxidant system and differential responses of SkM to a single bout of exercise compared to multiple sets of short-term exercise training.

Intracellular redox homeostasis is crucial for maintaining normal muscle structure and function. However, this balance tends to be disturbed during exercise due to increased ROS production by membrane NADPH oxidases and mitochondria, where the oxidative phosphorylation process increases dramatically to meet the increased ATP demand. As shown in Panel A of Figure 4, we detected a profound increase in ROS in mitochondria, cytosol, and whole cells receiving 4 days of EPS. The exact mechanisms responsible for this increased ROS remain to be determined. However, given the data in Panels A and B of Figure 5, a significant upregulation of complexes I/V and an increased mitochondrial oxygen consumption rate were detected. We believe that mitochondria are, at least, one of the major sources of the increased ROS in these EPS cells [34].

To preserve redox homeostasis in response to oxidative stress, intracellular antioxidant defenses must be mobilized to remove the excessive ROS. Antioxidant protein expression is primarily orchestrated by the Nrf2-Keap1 system, a thiol-based sensor-effector apparatus, where ROS oxidizes three key cysteine residues (Cys151, Cys273, and Cys288) on the Keap1 molecule, resulting in dissociation of Nrf2 from Keap1 and translocation from cytosol to the nucleus. In the nucleus, Nrf2 binds to antioxidant response elements (AREs) on DNA to upregulate hundreds of proteins involved in antioxidant defense, anti-inflammation, detoxification, and metabolism [10]. This ROS-trigger and Nrf2-Keap1-dependent activation of antioxidant defense in SkM has been characterized by our laboratory by employing transgenic mouse models with muscle-specific Nrf2/Keap1 deficiency, iMS-*Nrf2^flox/flox^* and iMS-*Keap1^flox/flox^* [11], and in a chronic exercise training model in C57BL/6 mice [12]. In the present study, using 4 days of EPS on C2C12 myotubes, we describe a useful in vitro model whose Nrf2/antioxidant system can be activated to a similar level as observed in animal models. This is particularly true for the expression of NQO1 and GSTA2 proteins.

One of the most important findings from the present study, we believe, is the prolonged elevated protein expression of antioxidant enzymes for up to 24 h after ceasing 4-day-EPS (Panel A, Figure 6). This phenomenon suggests potential cumulative effects on protein expression that are induced by consecutive multiple sets of EPS when the interval of any two chronologically adjacent stimulations is less than 24 h. This cumulative effect may represent a mechanism for the gradually increased protein contents of NQO1 and GSTA2, which were observed in the time-course experiment shown in Panel B of Figure 1. The translation of this in vitro experimental data into a strategy to optimize long-term exercise thus points to the significance of intervals between two sets of exercise-induced muscle antioxidant adaptation. While the molecular mechanisms underlying this prolonged antioxidant protein overexpression remain to be elucidated, we postulate that they are involved in an extended alteration of DNA transcription, protein translation, and/or post-translational modifications.

**Figure 7:**
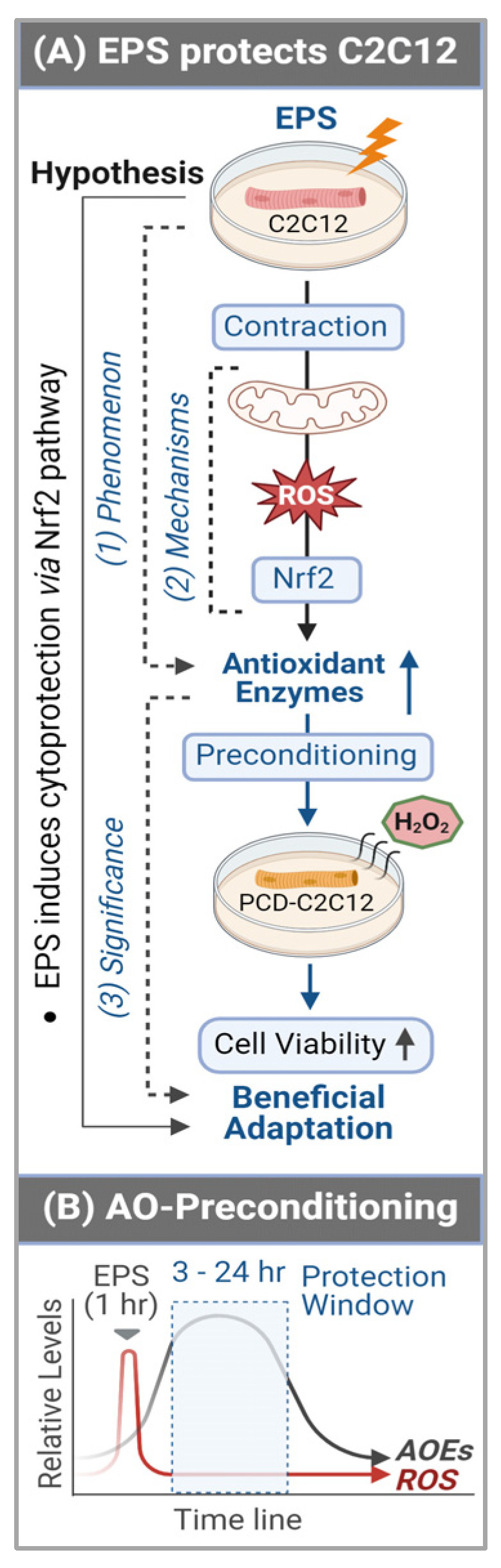
Graphical summary (**A**) and antioxidant preconditioning (**B**). AOEs—antioxidant (AO) enzymes (E); PCD—preconditioning.

In contrast to the expression of antioxidant enzymes, the EPS-induced increase in ROS did not last more than 3 h after ceasing stimulation (Panel B, Figure 6). This chronological dissociation between antioxidant enzymes and ROS tilts the redox balance to the antioxidant side and confers cells with excess and chronic antioxidant capacity—a state we term “antioxidant preconditioning” (Panel B, Figure 7)—thus protecting cells against subsequent oxidative injury. Indeed, as the data shows in Panel C of Figure 6, the EPS cells have 11- and 36-fold higher viability than the control cells when they are exposed to the medium containing 2 mM and 4 mM H_2_O_2_.

## 5. Conclusions

By employing C2C12 myotubes, we demonstrated that consecutive multiple sets of EPS over 4 days profoundly elevated the protein expression of a group of antioxidant enzymes when the interval of any two chronologically adjacent stimulations was less than 24 h. The translation of this in vitro experimental data into a strategy to optimize long-term exercise thus points to the significance of intervals between two sets of exercise-induced muscle antioxidant adaptation. This study provides evidence to demonstrate that short-term contraction can protect skeletal myocytes against subsequent oxidative stress-induced cytotoxicity, likely via ROS-dependent Nrf2-evoked activation of intracellular antioxidant mechanisms (Panel A, Figure 7), which provides a novel insight into the area of SkM redox signaling responses to exercise [34].

## Data Availability

The data are contained within the article.

## Abbreviations

CAT, catalase; GPX1, glutathione peroxidase 1; GSTA2, glutathione S-transferase alpha 2; GSTA4, glutathione S-transferase alpha 4; NQO1, NAD(P)H dehydrogenase [quinone] 1; Prx1, paired related homeobox 1; SOD1, superoxide dismutase type 1; SOD2, superoxide dismutase type 2; Trx, thioredoxin; TrxR1, thioredoxin reductase 1.
